# Open-Source Sequence Clustering Methods Improve the State Of the Art

**DOI:** 10.1128/mSystems.00003-15

**Published:** 2016-02-09

**Authors:** Evguenia Kopylova, Jose A. Navas-Molina, Céline Mercier, Zhenjiang Zech Xu, Frédéric Mahé, Yan He, Hong-Wei Zhou, Torbjørn Rognes, J. Gregory Caporaso, Rob Knight

**Affiliations:** a Department of Pediatrics, UCSD School of Medicine, La Jolla, California, USA; b Department of Computer Science and Engineering, University of California, San Diego, La Jolla, California, USA; c Laboratoire d'Ecologie Alpine (LECA), CNRS UMR 5553, Université Grenoble Alpes, Grenoble, France; d Department of Ecology, University of Kaiserslautern, Kaiserslautern, Germany; e Department of Environmental Health, State Key Laboratory of Organ Failure Research, Guangdong Provincial Key Laboratory of Tropical Disease Research, School of Public Health and Tropical Medicine, Southern Medical University, Guangzhou, Guangdong, China; f Department of Informatics, University of Oslo, Oslo, Norway; g Department of Microbiology, Oslo University Hospital, Rikshospitalet, Oslo, Norway; h Department of Biological Sciences, Northern Arizona University, Flagstaff, Arizona, USA; University of Trento

**Keywords:** sequence clustering, operational taxonomic units, microbial community analysis, amplicon sequencing

## Abstract

Sequence clustering is a common early step in amplicon-based microbial community analysis, when raw sequencing reads are clustered into operational taxonomic units (OTUs) to reduce the run time of subsequent analysis steps. Here, we evaluated the performance of recently released state-of-the-art open-source clustering software products, namely, OTUCLUST, Swarm, SUMACLUST, and SortMeRNA, against current principal options (UCLUST and USEARCH) in QIIME, hierarchical clustering methods in mothur, and USEARCH’s most recent clustering algorithm, UPARSE. All the latest open-source tools showed promising results, reporting up to 60% fewer spurious OTUs than UCLUST, indicating that the underlying clustering algorithm can vastly reduce the number of these derived OTUs. Furthermore, we observed that stringent quality filtering, such as is done in UPARSE, can cause a significant underestimation of species abundance and diversity, leading to incorrect biological results. Swarm, SUMACLUST, and SortMeRNA have been included in the QIIME 1.9.0 release.

**IMPORTANCE** Massive collections of next-generation sequencing data call for fast, accurate, and easily accessible bioinformatics algorithms to perform sequence clustering. A comprehensive benchmark is presented, including open-source tools and the popular USEARCH suite. Simulated, mock, and environmental communities were used to analyze sensitivity, selectivity, species diversity (alpha and beta), and taxonomic composition. The results demonstrate that recent clustering algorithms can significantly improve accuracy and preserve estimated diversity without the application of aggressive filtering. Moreover, these tools are all open source, apply multiple levels of multithreading, and scale to the demands of modern next-generation sequencing data, which is essential for the analysis of massive multidisciplinary studies such as the Earth Microbiome Project (EMP) (J. A. Gilbert, J. K. Jansson, and R. Knight, BMC Biol 12:69, 2014, http://dx.doi.org/10.1186/s12915-014-0069-1).

## Abstract (precis)

Massive collections of next-generation sequencing data call for fast, accurate, and easily accessible bioinformatics algorithms to perform sequence clustering. A comprehensive benchmark is presented, including open-source tools and the popular USEARCH suite. Simulated, mock, and environmental communities were used to analyze sensitivity, selectivity, species diversity (alpha and beta), and taxonomic composition. The results demonstrate that recent clustering algorithms can significantly improve accuracy and preserve estimated diversity without the application of aggressive filtering. Moreover, these tools are all open source, apply multiple levels of multithreading, and scale to the demands of modern next-generation sequencing data, which is essential for the analysis of massive multidisciplinary studies such as the Earth Microbiome Project (EMP) (J. A. Gilbert, J. K. Jansson, and R. Knight, BMC Biol 12:69, 2014, http://dx.doi.org/10.1186/s12915-014-0069-1).

## INTRODUCTION

Current DNA sequencing technologies generate hundreds of gigabytes of data in a single run and have enabled new detailed investigations into the human microbiome (1–3) and initiatives to characterize the Earth ecosystem’s microbiome, such as the EMP. Analysis of microbiome datasets typically begins by clustering raw biological sequence reads into operational taxonomic units (OTUs) based on sequence similarity, a process frequently referred to as OTU clustering or delineating. Sequencing costs are dropping faster than Moore’s law (4), increasing the need for efficient and accurate OTU clustering software. QIIME (5) has been using UCLUST (6) as the default clustering method since UCLUST’s publication (corresponding to QIIME version 1.0.0), due to its increase in performance over other popular tools, such as BLAST (7), DOTUR (8), or CD-HIT (9–11). However, UCLUST and USEARCH are closed-source software (the 64-bit versions, which are needed to handle large datasets, require an expensive license, even for academic use) and have limited documentation (http://www.drive5.com/usearch/). Moreover, based on UCLUST documentation (http://www.drive5.com/uclust/uclust_userguide_1_1_579.pdf), the allegedly serial implementation is impractical for massive high-throughput sequencing data. More accurate, faster, community-accessible tools are needed to overcome these challenges.

Within the previous 2 years, four new sequence-clustering tools have emerged: OTUCLUST from the Micca package (12), Swarm (13, 14), SUMACLUST (C. Mercier, F. Boyer, E. Kopylova, P. Taberlet, A. Bonin, and E. Coissac, submitted for publication), and SortMeRNA (15). These tools include open-source implementation, and the latter three implement multilevel parallelization, providing excellent potential alternatives to UCLUST. In this study, we evaluated these new open-source tools and compared them against UCLUST and USEARCH, two commonly used options available in QIIME, UPARSE (16), the latest USEARCH amplicon analysis pipeline, and the three hierarchical clustering algorithms available in mothur (17).

## RESULTS

### Software description.

OTU clustering can be performed in three different ways (18): closed reference, *de novo*, and open reference. In the closed-reference approach, the input sequences are clustered against a reference sequence database. In *de novo* clustering, the input sequences are grouped based on pairwise similarity among all sequences in the data set. The open-reference approach (19) begins by running a closed-reference step, which is followed by a *de novo* step that clusters the sequences that fail closed-reference assignment.

Swarm (13, 14) is a *de novo* clustering algorithm based on an unsupervised single-linkage-clustering method that reduces the impact of clustering parameters on the resulting OTUs by avoiding arbitrary global clustering thresholds and input sequence ordering dependence. Swarm builds OTUs in two steps: (i) an initial set of OTUs is constructed by iteratively agglomerating similar amplicons, and (ii) amplicon abundance values are used to reveal OTUs’ internal structures and to break them into sub-OTUs, if necessary.

OTUCLUST (12) and SUMACLUST are also *de novo* clustering algorithms; both are based on a greedy strategy in which the clusters are constructed incrementally by comparing an abundance-ordered list of input sequences against the representative set of already-chosen sequences (initially empty) (20). A similar approach is also used by UCLUST and CD-HIT, but OTUCLUST and SUMACLUST have been designed to perform exact sequence alignment, rather than relying on fast heuristics. In addition, OTUCLUST performs its own sequence dereplication and chimera removal (via UCHIME [21]).

mothur (17) implements three *de novo* clustering algorithms (nearest neighbor, furthest neighbor, and average neighbor) which cluster sequences based on genomic distance. In nearest neighbor (single linkage), a sequence is linked to an OTU if it is similar to any other sequence in that OTU, in furthest neighbor (complete linkage), a sequence is linked to an OTU if it is similar to all other sequences in that OTU, and in average neighbor, a sequence is linked to an OTU if it is similar to the averaged differences between all other sequences in that OTU. More details on these algorithms are available in references 8 and 22.

SortMeRNA (15) is suited for closed-reference OTU clustering. It is a local sequence alignment tool, in that it searches for optimal regions of similarity between two sequences. Query sequences (e.g., rRNA amplicons) are searched against a reference database, and an *E* value threshold is applied to evaluate the quality of resulting alignments. In SortMeRNA 2.0, the reference sequence achieving the lowest *E* value when aligned with a query sequence is chosen as the OTU centroid for that query. In addition to passing the *E* value threshold, the query must also have sufficient percent identity and coverage (both set to 97% by default). Contrary to UCLUST, the run time of SortMeRNA is not affected by reducing these thresholds (e.g., clustering at 60% identity).

UCLUST and USEARCH (versions 5.2 and 6.1) are supported in QIIME (v1.8.0). Both tools can perform *de novo*, closed-reference, and open-reference (except for USEARCH 5.2) clustering. In QIIME’s implementation, USEARCH 5.2 is executed via a pipeline closely shadowing otupipe (6, 21) to cluster OTUs, and USEARCH 6.1 performs chimera checking in an external script. UPARSE (16) is the latest *de novo* amplicon analysis pipeline from USEARCH; it applies stringent quality filtering and length trimming to remove erroneous reads and implements a novel greedy algorithm that performs OTU clustering and chimera removal concurrently.

### Experimental design.

Swarm 1.2.19, SUMACLUST 1.0.00, and SortMeRNA 2.0 have been integrated into QIIME 1.9.0 and can be used through QIIME’s three different OTU clustering commands (18): pick_closed_reference_otus.py, pick_de_novo_otus.py, and pick_open_reference_otus.py.

A variety of datasets were chosen to evaluate the performance of these open-source OTU clustering approaches relative to QIIME’s UCLUST/USEARCH-based OTU clustering approaches as well as UPARSE (see Table 1 for details). Two 16S rRNA gene simulated datasets were generated as FASTQ files. The first one (sim_even) represents an even distribution of 1,076 species, randomly subsampled from the Greengenes 97% database and computationally amplified at the same depth (100 reads/amplicon) and length (150 bp) using PrimerProspector (23) for extracting the V4 region and the ART (24) simulator for amplification and sequencing simulation. The second data set (sim_staggered) represents the same 1,076 species as the sim_even data set but amplified at different (random) species abundance levels. We used four different previously published mock community data sets: three 16S rRNA gene mock community data sets (Bokulich_2, Bokulich_3, and Bokulich_6) from Bokulich et al. (25) and an 18S gene (mock_nematodes) data set from Porazinska et al. (26). Finally, we also used three previously published natural data sets: a 16S rRNA gene soil data set (canadian_soil) from Neufeld et al. (27), a 16S rRNA gene human data set (body_sites) from Costello et al. (28), and an 18S rRNA gene soil data set (global_soil) from Ramirez et al. (29).

**TABLE 1:** Description of studies used in analysis

Data set	QIIME identity	Reference	Gene	Region	No. of reads	No. of samples	Read length	Platform
Simulated								
sim_even		24	16S	V4	107,600	1	150	ART
sim_staggered		24	16S	V4	107,025	1	150	ART
Mock								
Bokulich_2	1685	25	16S	V4	6,938,836	4	189–251	MiSeq
Bokulich_3	1686	25	16S	V4	3,594,237	4	114–151	MiSeq
Bokulich_6	1688	25	16S	V4	250,903	1	114–150	MiSeq
mock_nematodes		26	18S	V4	9,061	1	54–305	GS FLX
Genuine								
canadian_soil	632	27	16S	V4	2,966,053	13	76–100	HiSeq
body_sites	449	28	16S	V2	886,630	602	117–351	GS FLX
global_soil	2107	29	18S	V9	9,252,764	57	119–151	HiSeq

### Performance.

All tools were run with default parameters. Input FASTA files for Swarm, SUMACLUST, and SortMeRNA were generated using QIIME’s demultiplexing and quality filtering workflow. Input FASTA files for OTUCLUST, mothur, and UPARSE were demultiplexed using QIIME and quality filtered using each tool’s recommended standard operation procedure (SOP) (see Materials and Methods). Sequence filtering for OTUCLUST was performed with default quality score cutoffs of 20 (labeled as OTUCLUST_q20) and 3 (default in QIIME based on the results reported in reference 25, labeled as OTUCLUST_q3). UPARSE was run using the recommended settings with truncation lengths of 150 bp and 250 bp; similarly to OTUCLUST, runs were performed with a default quality score cutoff of 16 (labeled as UPARSE_q16) and additionally with a quality score cutoff of 3 (labeled as UPARSE_q3). Biological observation matrix (BIOM) format (30) tables were used as input to post clustering analyses. Taxonomy for reported OTUs was assigned using the RDP Classifier (31) against the 97% representative databases for Greengenes (32, 33) (version 13.8) and Silva (34) (version 111) for all methods. Performance was evaluated using a variety of metrics, including the accuracy of OTU and taxonomic assignments, alpha diversity (within-sample diversity), beta diversity (between-sample diversity), and taxonomic correlation. All tools showed increased precision after the removal of singleton OTUs (OTUs consisting of only one sequence), so all results presented here have had singleton OTUs removed. Table 2 summarizes basic performance results for all software.

**TABLE 2:**
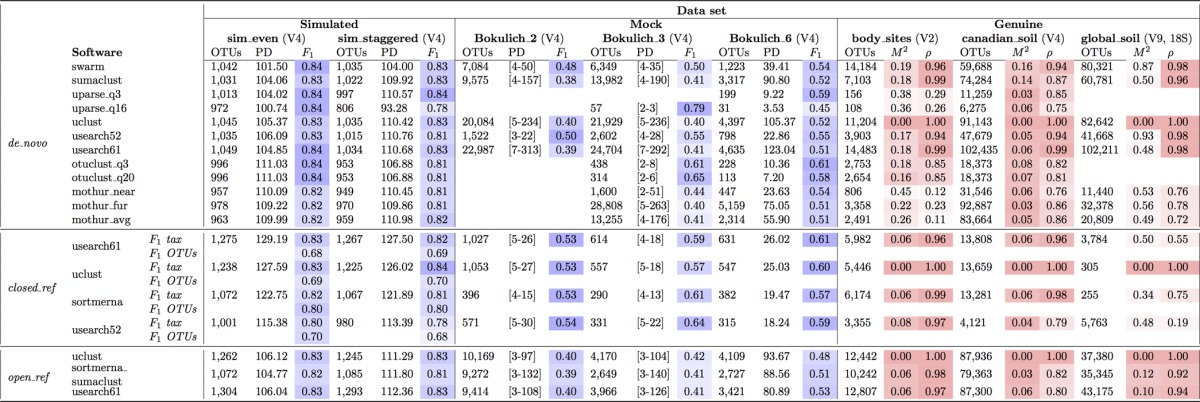
Benchmark summary^a^

a OTU counts do not include singletons. F measure (F1) is for assigned taxonomies at the genus level. The phylogenetic diversity (PD) whole-tree column for Bokulich_2 and Bokulich_3 represent PD intervals across various sampling depths. Procrustes M^2^ (the sum of the squared deviations or the dissimilarity of two datasets for UniFrac PCoA) and rho (Pearson’s correlation coefficient for taxonomies at genus level) values are with respect to UCLUST (default for QIIME versions 1.0.0 to 1.9.1). Monte Carlo *P* values were not included, since all values were <0.05 except for *de novo* usearch52 versus uclust (*P* = 0.09). The darkest blue shades represent the highest F1 scores, while the darkest red shades represent results closest to those obtained with UCLUST.

### Expected community composition: sensitivity and specificity. (i) Simulated data.

For *de novo* clustering, most tools report F measures (or F1 score, a metric that assesses the accuracy of taxonomic composition and observed OTUs, with a range from 0 to 1, where 1 is the best score) of 0.82 to 0.84 (sim_even) and 0.81 to 0.83 (sim_staggered) at the genus level (Table 2). Variation in results was emphasized in sim_staggered, where UPARSE_q16 reported the lowest F measure (0.78) as a result of stringent read filtering that removed nearly 95% of the reads prior to clustering. UPARSE_q3 removed roughly 4% of the reads and reported improved results on a par with those of Swarm, SUMACLUST, UCLUST, and USEARCH61. The highest F measure (0.83 to 0.84) and number of OTUs closest to the expected one (1,076) were reported for software using input files from QIIME’s method of sequence filtering (Swarm, SUMACLUST, UCLUST, and USEARCH61). All tools except UPARSE_q16 reported comparable alpha diversity phylogenetic diversity (PD) whole-tree (35) (a measure of diversity which considers the phylogenetic differences between species) values (mean of 109.21 with a standard deviation of 2.16 [Table 2]).

Among the closed-reference methods, SortMeRNA yields the fewest OTUs while achieving comparable or higher F measures for assigned taxonomy (F1 tax in Table 2) and OTUs (F1 OTUs in Table 2) and reported a phylogenetic diversity (121.89) closest to the ground truth (123.75) in comparison to UCLUST (126.02), USEARCH 5.2 (113.39), and USEARCH 6.1 (127.50). This is a result of SortMeRNA’s more exhaustive search for better alignments, which can increase run time but becomes imperative when short reads are aligned against a highly conservative set of sequences, such as the rRNA gene. The complete Greengenes database contains over a million rRNAs, and almost 73% of all full-length V4 regions (~250 nucleotides) are not unique. This emphasizes the highly conservative nature of rRNA, even in this hypervariable region, and suggests the need for thorough searches to ensure higher-quality alignments (especially for read lengths that do not cover the entire region). At the genus level of taxonomy, all tools report F measures of 0.80 to 0.83 (sim_even) and 0.78 to 0.84 (sim_staggered), which can be attributed to the many-to-one relationship between OTUs and taxonomy strings for the Greengenes 97% database.

For open-reference clustering, QIIME’s subsampling pipeline combining SortMeRNA and SUMACLUST reports the fewest OTUs in comparison to UCLUST and USEARCH 6.1. The F measure is 0.82 to 0.83 (sim_even) and 0.81 to 0.83 (sim_staggered) for all tools, which is in agreement with *de novo* and closed-reference results. These results are expected given the nature of open-reference clustering, which combines the closed-reference approach with the *de novo* approach.

### (ii) Mock communities.

Results for Bokulich_2 (and Bokulich_3 for UPARSE) are unavailable for UPARSE, OTUCLUST, and mothur due to significant memory, run time, and disk space requirements, respectively. All other methods were compared against the expected taxonomic composition for each data set. In addition, Pearson’s correlation coefficient was computed to measure the relatedness of taxonomic assignment between all pairs of tools (see column rho in Table 2 for all tools versus UCLUST). Values can range between −1 and 1, with −1 indicating a negative correlation, 0 indicating no correlation, and 1 indicating a positive correlation (strong relationship).

**FIG 1:**
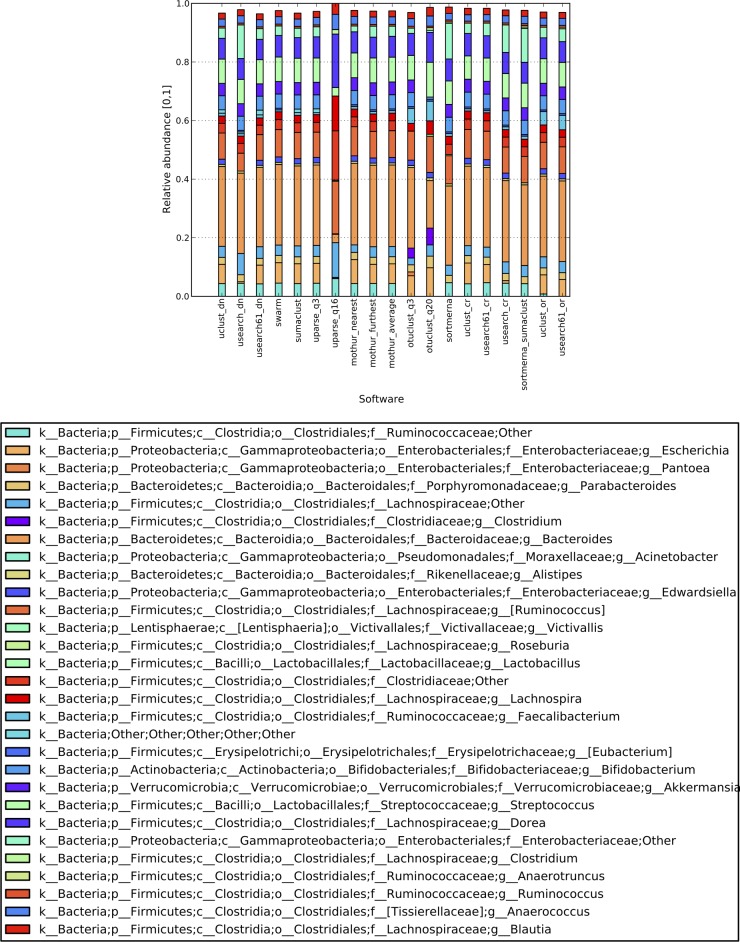
Layered bar chart showing top 20 abundant genera, Bokulich_6. The bars do not reach 1, since only a fraction (top 20) of taxonomies was illustrated.

For *de novo* clustering, USEARCH 5.2, UPARSE_(q3, q16), OTUCLUST_(q3, q20), and mothur_nearest frequently reported the lowest number of OTUs, the lowest number of observed taxa, and the highest F measure (Table 2). Since the F measure is computed using true-positive taxonomies based on the expected composition, possible contamination species (false positives) are unaccounted for. However, false-positive taxonomies can also arise from OTUs formed by chimeric sequences (sequences from two organisms that bind together during PCR and are subsequently sequenced as a single read) or incorrect assignment by taxonomy assignment tools. To investigate the origins of false-positive taxonomies reported by the tools, we checked all OTUs for chimeras using UCHIME (20) and mapped the nonchimeric OTUs against BLAST’s NT database using MEGABLAST. Most of the false-positive taxa were not wholly comprised of chimeric OTUs (meaning that the collection of OTUs mapping to the same taxa was composed of chimeric and genuine sequences), and the majority of such nonchimeric taxa consisted of OTUs mapping with an *E* value of <1e−50 to BLAST’s NT database (e.g., in Table 3 there are 57 false-positive taxa reported by SUMACLUST, but only 4 of those taxa are fully comprised of chimeric OTUs [FP-chimeric], and 99% of OTUs representing the remaining 53 nonchimeric taxa mapped with high similarity to BLAST’s NT database). Not surprisingly, all false-positive taxa whose OTUs mapped with <97% similarity (FP-other) are less abundant than the taxa whose OTUs map with ≥97% similarity (FP-known) and significantly less abundant than true-positive taxa (see Fig. S1 to S3 in the supplemental material). In fact, false-positive taxonomies (especially FP-other and FP-chimeric) comprise few and low-abundance OTUs, which can be analyzed and filtered out if necessary after clustering. Since UPARSE filters out a large fraction of presumably erroneous reads (even prior to chimera checking), it can detect the most abundant species (as can other tools) but also potentially overlook low-abundance species. For the Bokulich_2 and Bokulich_3 data sets, the top 20 most abundant genera follow a similar relative abundance distribution for all *de novo* tools, which is a direct reflection of hundreds of thousands of reads representing each expected genus in these data sets. However, for the much smaller data set Bokulich_6, UPARSE_q16 reported only half of the expected genera relative to all other tools, and the relative abundance of some of the genera significantly decreased (Fig. 1; PD values in Table 2). OTUCLUST, mothur_nearest, mothur_average, Swarm, and SUMACLUST reported significantly fewer OTUs than UCLUST and USEARCH 6.1, as well as a lower alpha diversity (Tables 2 and 3). Thus, tools with lower false-positive rates accomplish this by more stringent quality control, but they are less suitable for finding lower-abundance genera.

**TABLE 3:** Sensitivity and selectivity statistics for assigned taxonomies at genus level, Bokulich_2^a^

Software	No. of OTUs (no singletons)	P	R	F1	No. of taxonomies					
					TP	FN	FP			
							Total	Chimeric	Known	Other
*De novo*	**	**	**	**	**	**	**	**	**	**
usearch52	1,522	0.34	1	0.5	18	0	35	5	13	17
Swarm	7,084	0.32	1	0.48	18	0	38	7	22	9
uclust	20,084	0.25	1	0.4	18	0	53	4	15	34
usearch61	22,987	0.24	1	0.39	18	0	56	4	18	34
sumaclust	9,575	0.24	1	0.38	18	0	57	4	15	38
Closed reference										
usearch52	571	0.37	1	0.54	18	0	30	3	13	14
sortmerna	396	0.36	1	0.53	18	0	31	4	26	1
uclust	1,053	0.36	1	0.53	18	0	32	6	26	0
usearch61	1,027	0.36	1	0.53	18	0	32	4	28	0
Open reference										
uclust	10,169	0.25	1	0.4	18	0	52	4	19	29
usearch61	9,414	0.25	1	0.4	18	0	53	4	18	31
sortmerna_sumaclust	9,272	0.24	1	0.39	18	0	55	5	16	34

a P, precision; R, recall; F1, F measure, TP, true positive; FN, false negative; FP, false positive. The last three columns represent a refined breakdown of FP data, including false-positive taxonomies for which all comprising OTUs were classified as chimeric (using UCHIME) (chimeric), mapped to BLAST’s NT database with ≥97% similarity (known), or mapped to BLAST’s NT database with <97% similarity (other).

10.1128/mSystems.00003-15.1 Figure S1  Read abundance for true-positive and false-positive assigned taxonomies (order: *de novo*, closed reference, open reference), Bokulich_2. Download Figure S1, PDF file, 0.1 MB. Copyright © 2016 Kopylova et al. 2016 Kopylova et al. This content is distributed under the terms of the Creative Commons Attribution 4.0 International license.

10.1128/mSystems.00003-15.2 Figure S2  Read abundance for true-positive and false-positive assigned taxonomies (order: *de novo*, closed reference, open reference), Bokulich_3. Download Figure S2, PDF file, 0.1 MB. Copyright © 2016 Kopylova et al. 2016 Kopylova et al. This content is distributed under the terms of the Creative Commons Attribution 4.0 International license.

10.1128/mSystems.00003-15.3 Figure S3  Read abundance for true-positive and false-positive assigned taxonomies (order: *de novo*, closed reference, open reference), Bokulich_6. Download Figure S3, PDF file, 0.1 MB. Copyright © 2016 Kopylova et al. 2016 Kopylova et al. This content is distributed under the terms of the Creative Commons Attribution 4.0 International license.

For closed-reference clustering, SortMeRNA reported up to 60% fewer OTUs and a PD of about half that of UCLUST and USEARCH 6.1 (Table 2). On the genus taxonomy level, USEARCH 5.2 reported a high F measure (due to a lower number of false-positive genera), but unlike all other tools, the majority of false-positive genera are composed of reads mapping with ≤97% identity and coverage to BLAST’s NT database (FP-other). In fact, all other tools filtered out a large portion of these false-positive reads due to insufficient identity matches to the reference database. The difference appears to be caused by USEARCH 5.2’s identity definition (which does not consider insertions or deletions), which scores alignments higher than other tools. SortMeRNA generates taxonomic profiles similar to those obtained with other tools, with a Pearson’s correlation coefficient of >0.93 (Fig. 1).

As expected for open-reference clustering, SortMeRNA combined with SUMACLUST reported fewer OTUs than UCLUST and USEARCH 6.1 while preserving high accuracy and lower alpha diversity for both the number of observed OTUs and the phylogenetic diversity. Specific details can be found in the supplemental material.

The Pearson coefficient for comparisons between all tools and methods remained relatively stable, from ~0.99 (Bokulich_2) to 0.97 to 1 (Bokulich_3) to 0.92 to 0.99 (Bokulich_6), showing a strong relationship between all algorithms. The coefficient was lower in the cases where the taxonomy could not be assigned (e.g., 0.0273 for SortMeRNA versus UCLUST for data set Bokulich_3) or significant filtering of sequences (e.g., 0.3719 for UCLUST versus UPARSE_q16 for data set Bokulich_6).

### Natural community composition.

Results for UPARSE_q4 and UPARSE_q16 are unavailable for the global_soil data set due to memory limitations in the 32-bit version of UPARSE and for OTUCLUST due to significant run time (limited to one thread).

**FIG 2:**
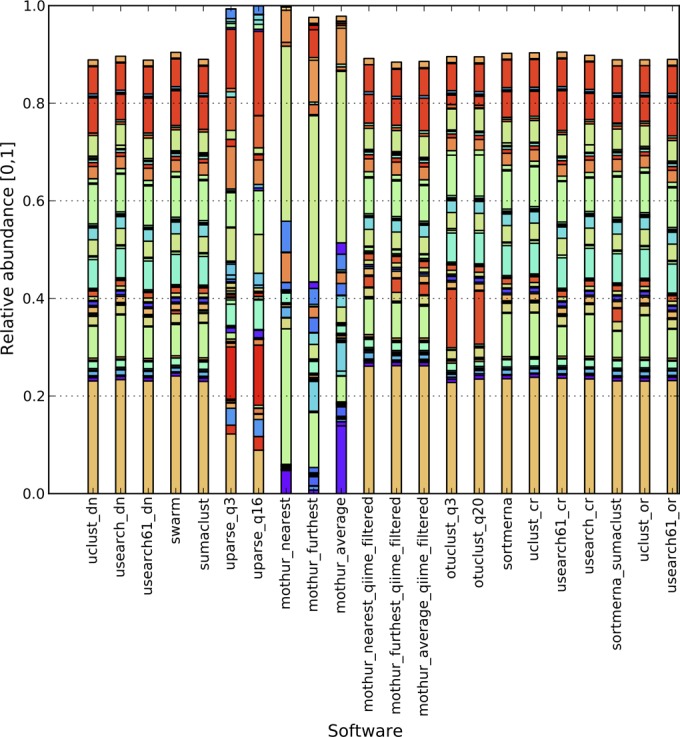

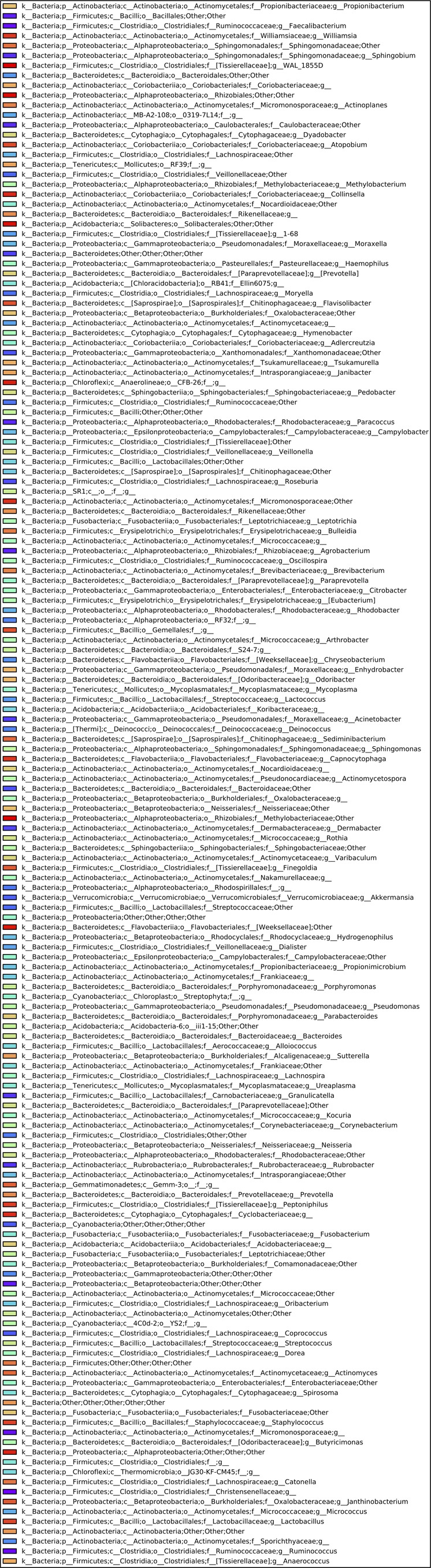
Taxonomic composition graph illustrating top 50 (per software) abundant genera, body_sites. The bars do not reach 1, since only a fraction (top 50) of taxonomies was illustrated. mothur was run using recommended filtering (trim.seqs function) for 454 SOP and with QIIME’s split_libraries_fastq.py to highlight the effect of different filtering methods.

**FIG 3:**
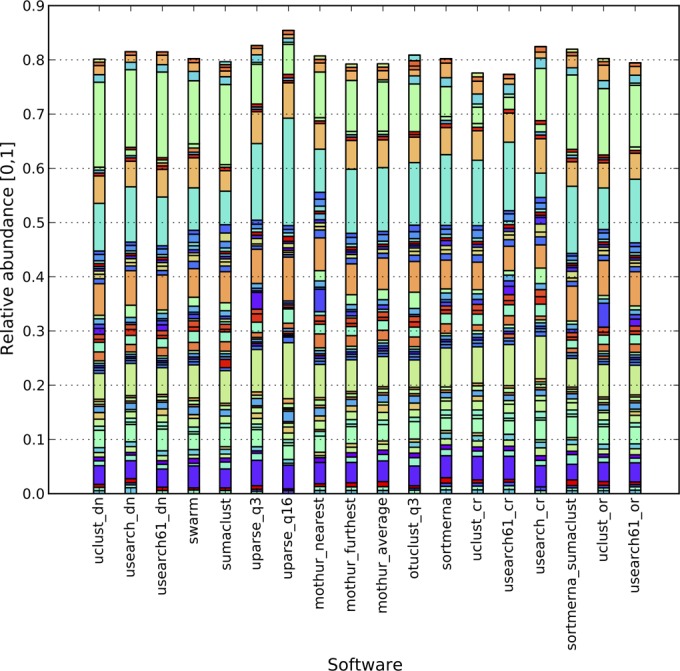

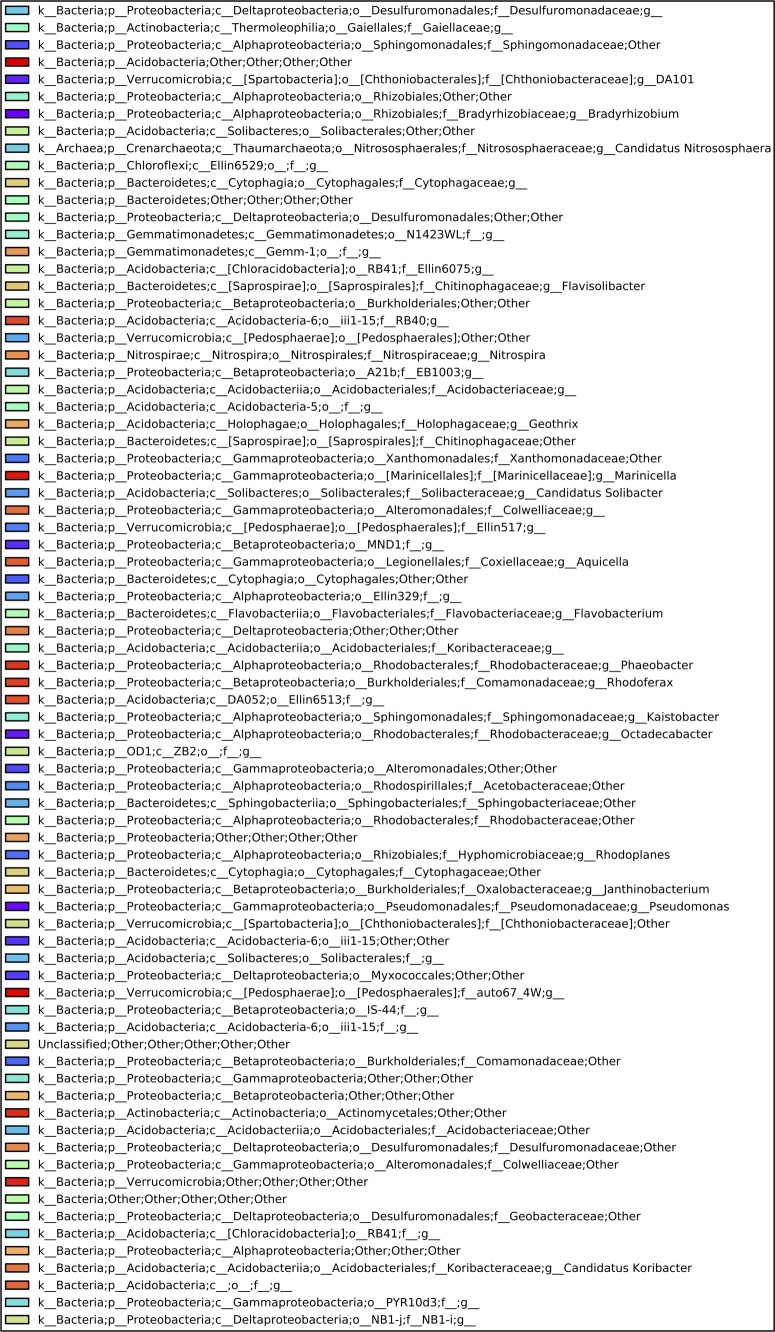
Taxonomic composition graph illustrating top 50 (per software) abundant genera, canadian_soil. The bars do not reach 1, since only a fraction (top 50) of taxonomies was illustrated.

In contrast to mock communities, the Pearson correlation for natural communities was much more variable (0.70 to 0.94 for the canadian_soil data set, 0.28 to 0.99 for the body_sites data set, and 0.19 to 0.98 for the global_soil data set) (Table 2), highlighting differences between all clustering algorithms in a complex environment that are not immediately visible in either simulated or mock communities. These ranges do not take into account outliers that were caused by an inconsistency with RDP assignments for the most abundant taxa (Bokulich_3) and stringent filtering of reads by UPARSE_q16 (Bokulich_6) (Fig. 1). QIIME, UPARSE, OTUCLUST, and mothur include different sequence filtering methods, which could be the major reason behind inconsistent taxonomic compositions (Pearson’s correlation in Table 2; Fig. 2). As illustrated in Fig. 2, running mothur with sequences that were quality-filtered by mothur and QIIME produced significantly different taxonomic compositions. As expected, the highest correlation exists for studies with the longest reads and the largest number of reads per sample, showing that clustering results converge to the same conclusions with longer, higher-quality reads and deep sequencing (Fig. 3).

**FIG 4:**
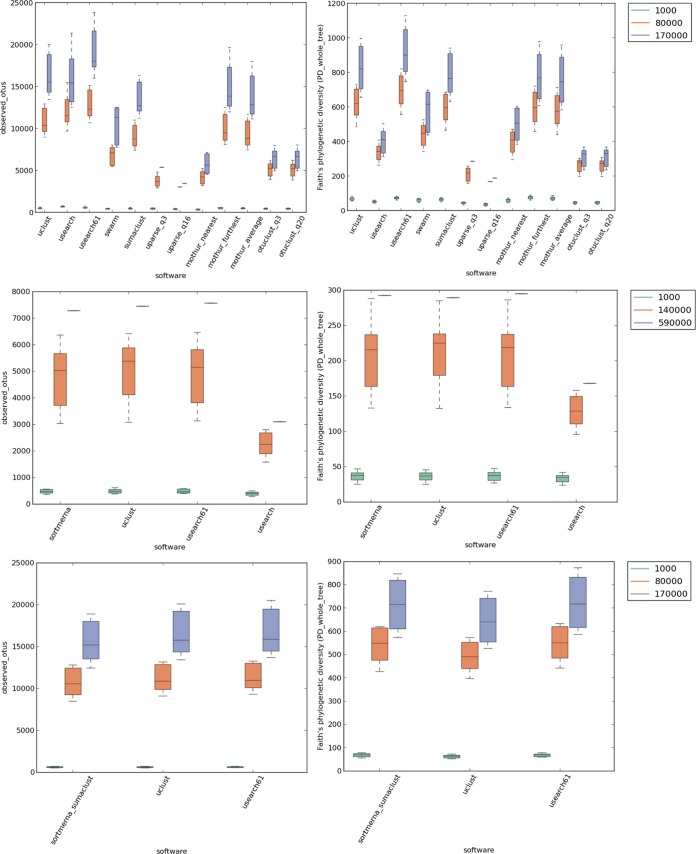
Alpha diversity for tools at different sampling depths (order: *de novo*, closed reference, and open reference), canadian_soil.

As with the mock-community results, all tools frequently reported fewer OTUs and lower alpha diversities than UCLUST and USEARCH 6.1 (Table 2 and Fig. 4; also, see Fig. S4 and S5 in the supplemental material). Procrustes analysis (36) was used to compare unweighted UniFrac (37, 38) principal coordinates analysis (PCoA) (22) generated by all methods versus UCLUST (the current default OTU picker in QIIME). The Procrustes M^2^ metric for body_sites and canadian_soil was <0.3 for most software (Table 2), indicating that beta diversity patterns are similar irrespective of the OTU clustering method used. Neither recommended nor relaxed quality filtering parameters for UPARSE worked well for the body_sites data set, where 98.5% and 99.2% of reads were filtered out for UPARSE_q3 and UPARSE_q16 (with a trim length of 250 bp), respectively, resulting in very few remaining samples and high M^2^ values (Table 2; also, see Fig. S4 in the supplemental material). Although read quality filtering is an important preprocessing step, more work is required to regulate these parameters (perhaps by an automated estimation of optimal truncation length and quality), as they can be very sensitive to different types of data.

10.1128/mSystems.00003-15.4 Figure S4  Alpha diversity for tools at different sampling depths (order: *de novo*, closed reference, open reference), body_sites. For *de novo* graphs, the legend represents various sampling depths. Download Figure S4, PDF file, 0.2 MB. Copyright © 2016 Kopylova et al. 2016 Kopylova et al. This content is distributed under the terms of the Creative Commons Attribution 4.0 International license.

10.1128/mSystems.00003-15.5 Figure S5  Alpha diversity for tools at different sampling depths (order: *de novo*, closed reference, open reference), global_soil. For *de novo* graphs, the legend represents various sampling depths. Download Figure S5, PDF file, 0.2 MB. Copyright © 2016 Kopylova et al. 2016 Kopylova et al. This content is distributed under the terms of the Creative Commons Attribution 4.0 International license.

## DISCUSSION

We evaluated the performance of four recently published open-source sequence clustering tools against the widely used mothur, UCLUST, and USEARCH tools using simulated data, mock communities, and natural microbial communities. We found that Swarm, SUMACLUST, UCLUST, and UPARSE (with relaxed parameters) performed equally well on simulated datasets where the ground truth was well established, with mothur_average and OTUCLUST closely behind. Despite this controlled chimera-free environment, UPARSE with recommended parameters reported the lowest accuracy for the sim_staggered data set, implying that stringent quality filtering can cause a significant underestimation of species abundance and diversity and lead to incorrect biological results. For the mock communities, most tools were able to correctly detect the expected number and identity of genera, but only UPARSE reported significantly fewer false-positive taxa (followed by OTUCLUST and USEARCH). For UPARSE, this was expected, as a large proportion of reads was filtered out prior to clustering, leaving evidence of only the most abundant taxa (OTUs comprised of hundreds of thousands of reads). The majority of false-positive taxa reported by other tools were low-abundance OTUs that could be mapped to BLAST’s NT database with very high similarity (*E* value, <1e−50). If the user’s primary goal is to focus on the most abundant microbial profiles, low-abundance OTUs may be filtered out postclustering, but care should be taken, as such low-abundance OTUs can be important members of communities (39).

In terms of accurately predicted taxonomic composition for *de novo* tools, Swarm performed well across all simulated and mock datasets, followed closely by SUMACLUST and UCLUST. However, both Swarm and SUMACLUST reported significantly fewer OTUs and lower alpha diversities than UCLUST. The performance of other *de novo* methods, such as mothur and OTUCLUST, showed more variation across datasets; however, these results were largely influenced by the preliminary sequence filtering step, where both tools removed more data than QIIME’s method. We found that QIIME’s filtering approach worked well across all datasets, rendering the most data for clustering tools to work with. For closed-reference tools, SortMeRNA significantly outperformed UCLUST and USEARCH for predicting OTUs and performed as well or better in terms of predicted taxonomic composition. Several studies could not be processed with mothur, OTUCLUST, or the free academic distribution of UPARSE due to their large sizes, either because of an unreasonable disk space requirement in the case of mothur, unreasonable run time in the case of OTUCLUST (no multithreading support), or a relatively small memory limit in the case of UPARSE. Regarding UPARSE, the small memory limit makes it necessary to purchase the 64-bit license in order to process large projects (e.g., see Yatsunenko et al.’s work [40], which contains 500 GB of raw sequence data generated on 17 HiSeq lanes) or use open-source alternatives. QIIME’s current open-source, open-reference pipeline (based on SortMeRNA and SUMACLUST) was able to process this quantity of data within 24 h using 64 threads on Intel Xeon CPU E5-4620 v2 at 2.60GHz or within 3 days using 64 threads on AMD Opteron Processor 6276.

**FIG 5:**
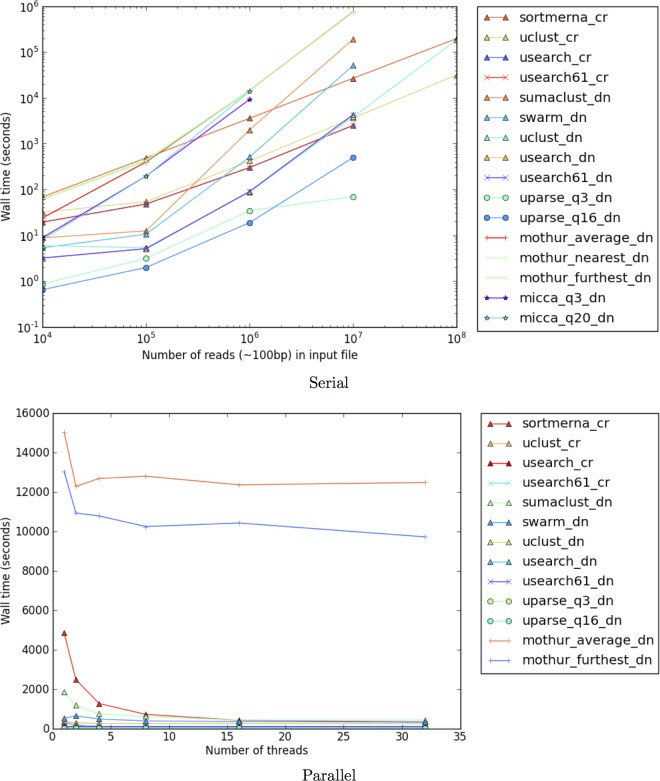
Run time performance for all benchmarked software. All tests were performed using 1 to 32 cores on Intel Xeon CPU E5-2640 v3 at 2.60 GHz. Input files contained reads subsampled from the Global Gut. For serial performance, some tools do not show results for 10^8^ reads due to exceeding wall time limit (230 h) or failed memory allocation. For parallel performance, a single file containing 1 million Illumina sequences was used over multiple threads.

Although most open-source tools report an increased run time in comparison to UCLUST and USEARCH (Fig. 5), they provide the benefit of finding significantly fewer OTUs. In the case of SortMeRNA, longer reads (~150 bp) are quicker to align than the same number of shorter reads (~100 bp) due to many fewer high-scoring candidate reference sequences to analyze. Moreover, all of these tools support multilevel multithreading and can easily scale to modern big-data processing demands. An alternative to reducing run time is to filter out a substantial number of reads, as done by UPARSE; unfortunately, the filtering parameters are sensitive to different data, and choosing them manually by trial and error can be a time-consuming task with unpredictable outcomes in diversity.

The three open-source software products, Swarm, SUMACLUST, and SortMeRNA, are now accessible through the widely used QIIME software package (release 1.9.0). Swarm 2 was released in reference 14 and reported to be faster and more memory efficient than Swarm 1; however, as of this writing, only Swarm 1 has been integrated into QIIME. Ongoing work to improve the QIIME OTU clustering workflows that use these tools includes adding a targeted gene prefilter for *de novo* clustering to remove (prior to clustering) any sequences not matching a specific gene model (e.g., 16S rRNA) and a refined reference database for targeted hypervariable regions (e.g., V4 at 97% identity) to improve alignment quality (41). Furthermore, research is in progress to introduce an open-source implementation of chimera detection directly within QIIME. Both of these improvements will further reduce the number of unrelated or erroneous reads recruited into OTUs, a known problem with both the UCLUST- and USEARCH-based OTU clustering illustrated here, without underestimating diversity.

## MATERIALS AND METHODS

All steps taken to generate the analyses presented in this article are documented and implemented as shell or python scripts, available at https://github.com/ekopylova/OTU-clustering.

### Performance benchmarks.

Open-source with multilevel parallelization tools tested in this paper—Swarm, SUMACLUST, and SortMeRNA—have been integrated into QIIME 1.9.0. For these tools, all benchmarks were launched through QIIME. For UPARSE, the recommended workflow (http://www.drive5.com/usearch/manual/uparse_cmds.html) was run. For OTUCLUST, the script micca-preproc was used for sequence filtering and the command otuclust for clustering. For mothur, the MiSeq SOP (42) (website accessed 27 October 2015) and 454 SOP (43) (website accessed 27 October 2015) were run. The shell scripts commands_16S.sh and commands_18S.sh were used to launch all tools, and the open-source project (https://github.com/josenavas/QIIME-Scaling) was used for measuring their run time performance. All run time performance tests were performed using 1 to 32 threads on Intel Xeon CPU E5-2640 v3 at 2.60 GHz.

### Precision and recall.

For simulated and mock datasets, false-positive (FP; taxonomy/OTU string exists in observed but not expected), false-negative (FN; taxonomy/OTU string exists in expected but not observed), and true-positive (TP; taxonomy/OTU string exists in both observed and expected) measures were computed between the pickers’ results (observed) and the ground truth or expected taxonomic composition (expected). The following definitions were used: precision = TP/(TP + FP); recall = TP/(TP + FN); F measure = 2 × precision × recall/(precision + recall).

The python script run_compute_precision_recall.py was used to compute TP, FP, FN, precision, recall, F measure, the number of false-positive taxa whose complete set of OTUs are identified as chimeric (FP-chimeric) by UCHIME, the number of false-positive taxa whose complete set of OTUs map with ≥97% identity and coverage to BLAST’s NT database (FP-known), and the number of false-positive taxa whose complete set of OTUs map with <97% identity and coverage to BLAST’s NT database (FP-other). The script plot_tp_fp_distribution.py was used to generate Fig. S1, S2, and S3 in the supplemental material.

### Simulating reads (even and staggered).

All of the following steps can be executed using the shell script simulate_reads.sh.

Reads were simulated using PrimerProspector (23) and the ART simulator (24). For the even data set, the following steps were taken. (i) Use PrimerProspector to extract V4 regions from the Greengenes 97% representative database (version 13.8); (ii) subsample 0.011% of the sequences from the resulting V4 region database; and (iii) simulate even abundance reads with ART simulator using the subsampled V4 sequences.

Amplicon sequencing simulation in ART (version VanillaIceCream-03-11-2014) could generate only evenly distributed communities. To simulate the staggered data set, a staggered distribution of template sequences was passed (for example, 3 duplicates of OTU1, 10 duplicates of OTU2, etc.). To simulate the staggered data set, the following steps were taken. (i) Generate a random staggered distribution FASTA file of template V4 sequences using the list of OTU identifications from the even data set and the V4 subsampled sequences and (ii) simulate staggered abundance reads with ART using the staggered subsampled V4 sequences.

For both even and staggered reads, QIIME’s split_libraries_fastq.py script was run to filter simulated reads based on quality scores and format FASTA labels to be compatible with QIIME (reads for UPARSE, mothur, and OTUCLUST were not filtered; only FASTA labels were reformatted).

### Building ground-truth BIOM tables.

Ground-truth OTU maps and BIOM tables were constructed using the simulate_reads.sh script that was used for simulating reads. OTU maps were generated using the reads’ origin information stored in the FASTA labels of ART-simulated reads. BIOM tables were generated using QIIME’s make_otu_table.py script together with Greengenes 97% taxonomy strings.

### Construction of a Silva 97% representative OTU tree.

A eukaryotic/18S rRNA sequence set tree was built using QIIME’s filter_alignment.py and make_phylogeny.py scripts:

filter_alignment.py -i Silva_111_post/rep_set_aligned/97_Silva_111_rep_set.fasta -e 0.0005 -g 0.80 -o Silva_111_post/trees; make_phylogeny.py -i Silva_111_post/trees/97_Silva_111_rep_set_pfiltered.fasta -o Silva_111_post/trees/97_Silva_111_rep_set_pfiltered.tre.

### Calculating alpha diversity, beta diversity, and taxonomic correlation.

Customs scripts iterating over all benchmarking results were used to launch QIIME’s alpha and beta diversity analyses. The script run_single_rarefaction_and_plot.py was used to compute and plot alpha diversity as shown in Fig. 4 and in Fig. S4 and S5 in the supplemental material. The script run_beta_diversity_and_procrustes.py was used to compute beta diversity and run Procrustes analysis.

10.1128/mSystems.00003-15.6 Table S1  URLs for software and studies used in this analysis. Download Table S1, PDF file, 0.04 MB. Copyright © 2016 Kopylova et al. 2016 Kopylova et al. This content is distributed under the terms of the Creative Commons Attribution 4.0 International license.

10.1128/mSystems.00003-15.7 Table S2  Expected genera for Bokulich_2 and Bokulich_3. Download Table S2, PDF file, 0.02 MB. Copyright © 2016 Kopylova et al. 2016 Kopylova et al. This content is distributed under the terms of the Creative Commons Attribution 4.0 International license.

10.1128/mSystems.00003-15.8 Table S3  Expected families for mock_nematodes. Download Table S3, PDF file, 0.02 MB. Copyright © 2016 Kopylova et al. 2016 Kopylova et al. This content is distributed under the terms of the Creative Commons Attribution 4.0 International license.

10.1128/mSystems.00003-15.9 Table S4  Expected genera for Bokulich_6. Download Table S4, PDF file, 0.02 MB. Copyright © 2016 Kopylova et al. 2016 Kopylova et al. This content is distributed under the terms of the Creative Commons Attribution 4.0 International license.

10.1128/mSystems.00003-15.10 Table S5  Sensitivity and selectivity statistics for assigned taxonomies at genus level, mock_nematodes. After software, columns represent OTU count (excluding singleton OTUs), precision (P), recall (R), F measure, TP (number of true-positive taxonomies), FN (number of false-negative taxonomies), FP (number of false-positive taxonomies). The remaining three FP columns represent a refined breakdown of the FP column, including false-positive taxonomies for which all comprising OTUs were classified chimeric (using UCHIME) (FP chimeric), mapped to BLAST’s NT database with ≥97% similarity (FP-known), or mapped to BLAST’s nt database with <97% similarity (FP-other). Download Table S5, PDF file, 0.04 MB. Copyright © 2016 Kopylova et al. 2016 Kopylova et al. This content is distributed under the terms of the Creative Commons Attribution 4.0 International license.

## References

[1] The Human Microbiome Project Consortium 2012 A framework for human microbiome research. Nature 486:215–221. doi:10.1038/nature11209. 22699610 PMC3377744

[2] Human Microbiome Project Consortium 2012 Structure, function and diversity of the healthy human microbiome. Nature 486:207–214. doi:10.1038/nature11234. 22699609 PMC3564958

[3] Turnbaugh PJ, Ley RE, Hamady M, Fraser-Liggett CM, Knight R, Gordon JI 2007 The human microbiome project. Nature 449:804–810. doi:10.1038/nature06244. 17943116 PMC3709439

[4] Wetterstrand KA 2013 DNA sequencing costs: data from the NHGRI genome sequencing program (GSP). http://www.genome.gov/sequencingcosts. Accessed 15 November 2014.

[5] Caporaso JG, Kuczynski J, Stombaugh J, Bittinger K, Bushman FD, Costello EK, Fierer N, Peña AG, Goodrich JK, Gordon JI, Huttley GA, Kelley ST, Knights D, Koenig JE, Ley RE, Lozupone CA, McDonald D, Muegge BD, Pirrung M, Reeder J, Sevinsky JR, Turnbaugh PJ, Walters WA, Widmann J, Yatsunenko T, Zaneveld J, Knight R 2010 QIIME allows analysis of high-throughput community sequencing data. Nat Methods 7:335–336. doi:10.1038/nmeth.f.303.7. 20383131 PMC3156573

[6] Edgar RC 2010 Search and clustering orders of magnitude faster than BLAST. Bioinformatics 26:2460–2461. doi:10.1093/bioinformatics/btq461. 20709691

[7] Altschul SF, Gish W, Miller W, Myers EW, Lipman DJ 1990 Basic local alignment search tool. J Mol Biol 215:403–410. doi:10.1006/jmbi.1990.9999. 2231712

[8] Schloss PD, Handelsman J 2005 Introducing DOTUR, a computer program for defining operational taxonomic units and estimating species richness. Appl Environ Microbiol 71:1501–1506. doi:10.1128/AEM.71.3.1501. 15746353 PMC1065144

[9] Li W, Jaroszewski L, Godzik A 2001 Clustering of highly homologous sequences to reduce the size of large protein databases. Bioinformatics 17:282–283. doi:10.1093/bioinformatics/17.3.282. 11294794

[10] Li W, Jaroszewski L, Godzik A 2002 Tolerating some redundancy significantly speeds up clustering of large protein databases. Bioinformatics 18:77–82. doi:10.1093/bioinformatics/18.1.77. 11836214

[11] Li W, Godzik A 2006 Cd-hit: a fast program for clustering and comparing large sets of protein or nucleotide sequences. Bioinformatics 22:1658–1659. doi:10.1093/bioinformatics/btl158. 16731699

[12] Albanese D, Fontana P, De Filippo C, Cavalieri D, Donati C 2015 Micca: a complete and accurate software for taxonomic profiling of metagenomic data. Sci Rep 5:9743. doi:10.1038/srep09743. 25988396 PMC4649890

[13] Mahé F, Rognes T, Quince C, de Vargas C, Dunthorn M 2014 Swarm: robust and fast clustering method for amplicon-based studies. PeerJ 2:e593. doi:10.7717/peerj.593. 25276506 PMC4178461

[14] Mahé F, Rognes T, Quince C, de Vargas C, Dunthorn M 2015 Swarm v2: highly-scalable and high-resolution amplicon clustering. PeerJ 3:e1420. doi:10.7717/peerj.1420. 26713226 PMC4690345

[15] Kopylova E, Noé L, Touzet H 2012 SortMeRNA: fast and accurate filtering of ribosomal RNAs in metatranscriptomic data. Bioinformatics 28:3211–3217. doi:10.1093/bioinformatics/bts611. 23071270

[16] Edgar RC 2013 UPARSE: highly accurate OTU sequences from microbial amplicon reads. Nat Methods 10:996–998. doi:10.1038/nmeth.2604. 23955772

[17] Schloss PD, Westcott SL, Ryabin T, Hall JR, Hartmann M, Hollister EB, Lesniewski RA, Oakley BB, Parks DH, Robinson CJ, Sahl JW, Stres B, Thallinger GG, Van Horn DJ, Weber CF 2009 Introducing mothur: open-source, platform-independent, community-supported software for describing and comparing microbial communities. Appl Environ Microbiol 75:7537–7541. doi:10.1128/AEM.01541-09. 19801464 PMC2786419

[18] Navas-Molina JA, Peralta-Sánchez JM, González A, McMurdie PJ, Vázquez-Baeza Y, Xu Z, Ursell LK, Lauber C, Zhou H, Song SJ, Huntley J, Ackermann GL, Berg-Lyons D, Holmes S, Caporaso JG, Knight R 2013 Advancing our understanding of the human microbiome using QIIME. Methods Enzymol 531:371–444. doi:10.1016/B978-0-12-407863-5.00019-8. 24060131 PMC4517945

[19] Rideout JR, He Y, Navas-Molina JA, Walters WA, Ursell LK, Gibbons SM, Chase J, McDonald D, Gonzalez A, Robbins-Pianka A, Clemente JC, Gilbert JA, Huse SM, Zhou H-W, Knight R, Caporaso JG 2014 Subsampled open-reference clustering creates consistent, comprehensive OTU definitions and scales to billions of sequences. PeerJ 2:e545. doi:10.7717/peerj.545. 25177538 PMC4145071

[20] Hobohm U, Scharf M, Schneider R, Sander C 1992 Selection of representative protein data sets. Protein Sci 1:409–417. doi:10.1002/pro.5560010313. 1304348 PMC2142204

[21] Edgar RC, Haas BJ, Clemente JC, Quince C, Knight R 2011 UCHIME improves sensitivity and speed of chimera detection. BioInformatics 27:2194–2200. doi:10.1093/bioinformatics/btr381. 21700674 PMC3150044

[22] Legendre P, Legendre L 1998 Numerical ecology, 2nd ed Developments in environmental modelling, vol 20, p 870 Elsevier Science, Amsterdam, The Netherlands.

[23] Walters WA, Caporaso JG, Lauber CL, Berg-Lyons D, Fierer N, Knight R 2011 PrimerProspector: de novo design and taxonomic analysis of barcoded polymerase chain reaction primers. Bioinformatics 27:1159–1161. doi:10.1093/bioinformatics/btr087. 21349862 PMC3072552

[24] Huang W, Li L, Myers JR, Marth GT 2012 ART: a next-generation sequencing read simulator. BioInformatics 28:593–594. doi:10.1093/bioinformatics/btr708. 22199392 PMC3278762

[25] Bokulich NA, Subramanian S, Faith JJ, Gevers D, Gordon JI, Knight R, Mills DA, Caporaso JG 2013 Quality-filtering vastly improves diversity estimates from Illumina amplicon sequencing. Nat Methods 10:57–59. doi:10.1038/nmeth.2276. 23202435 PMC3531572

[26] Porazinska DL, Giblin-Davis RM, Faller L, Farmerie W, Kanzaki N, Morris K, Powers TO, Tucker AE, Sung W, Thomas WK 2009 Evaluating high-throughput sequencing as a method for metagenomic analysis of nematode diversity. Mol Ecol Resour 9:1439–1450. doi:10.1111/j.1755-0998.2009.02611.x. 21564930

[27] Neufeld JD, Engel K, Cheng J, Moreno-Hagelsieb G, Rose DR, Charles TC 2011 Open resource metagenomics: a model for sharing metagenomic libraries. Stand Genomic Sci 5:203–210. doi:10.4056/sigs.1974654. 22180823 PMC3235511

[28] Costello EK, Lauber CL, Hamady M, Fierer N, Gordon JI, Knight R 2009 Bacterial community variation in human body habitats across space and time. Science 326:1694–1697. doi:10.1126/science.1177486. 19892944 PMC3602444

[29] Ramirez KS, Leff JW, Barberán A, Bates ST, Betley J, Crowther TW, Kelly EF, Oldfield EE, Shaw EA, Steenbock C, Bradford MA, Wall DH, Fierer N 2014 Biogeographic patterns in below-ground diversity in New York City’s Central Park are similar to those observed globally. Proc R Soc B Biol Sci 281:1–9. doi:10.1098/rspb.2014.1988. PMC4213626 25274366

[30] McDonald D, Clemente JC, Kuczynski J, Rideout JR, Stombaugh J, Wendel D, Wilke A, Huse S, Hufnagle J, Meyer F, Knight R, Caporaso JG 2012 The biological observation matrix (BIOM) format or: how I learned to stop worrying and love the ome-ome. Gigascience 1:7. doi:10.1186/2047-217X-1-7. 23587224 PMC3626512

[31] Wang Q, Garrity GM, Tiedje JM, Cole JR 2007 Naive Bayesian classifier for rapid assignment of rRNA sequences into the new bacterial taxonomy. Appl Environ Microbiol 73:5261–5267. doi:10.1128/AEM.00062-07. 17586664 PMC1950982

[32] DeSantis TZ, Hugenholtz P, Larsen N, Rojas M, Brodie EL, Keller K, Huber T, Dalevi D, Hu P, Andersen GL 2006 Greengenes, a chimera-checked 16S rRNA gene database and workbench compatible with ARB. Appl Environ Microbiol 72:5069–5072. doi:10.1128/AEM.03006-05. 16820507 PMC1489311

[33] McDonald D, Price MN, Goodrich J, Nawrocki EP, DeSantis TZ, Probst A, Andersen GL, Knight R, Hugenholtz P 2012 An improved Greengenes taxonomy with explicit ranks for ecological and evolutionary analyses of bacteria and archaea. ISME J 6:610–618. doi:10.1038/ismej.2011.139. 22134646 PMC3280142

[34] Pruesse E, Quast C, Knittel K, Fuchs BM, Ludwig W, Peplies J, Glöckner FO 2007 Silva: a comprehensive online resource for quality checked and aligned ribosomal RNA sequence data compatible with ARB. Nucleic Acids Res 35:7188–7196. doi:10.1093/nar/gkm864. 17947321 PMC2175337

[35] Faith DP 1992 Conservation evaluation and phylogenetic diversity. Biol Conserv 61:1–10. doi:10.1016/0006-3207(92)91201-3.

[36] Gower JC 1975 Generalized Procrustes analysis. Psychometrika 40:33–51. doi:10.1007/BF02291478.

[37] Lozupone C, Lladser ME, Knights D, Stombaugh J, Knight R 2011 UniFrac: an effective distance metric for microbial community comparison. ISME J 5:169–172. doi:10.1038/ismej.2010.133. 20827291 PMC3105689

[38] Hamady M, Lozupone C, Knight R 2010 Fast UniFrac: facilitating high-throughput phylogenetic analyses of microbial communities including analysis of pyrosequencing and PhyloChip data. ISME J 4:17–27. doi:10.1038/ismej.2009.97. 19710709 PMC2797552

[39] Shade A, Jones SE, Caporaso JG, Handelsman J, Knight R, Fierer N, Gilbert JA 2014 Conditionally rare taxa disproportionately contribute to temporal changes in microbial diversity. mBio 5:e01371-14. doi:10.1128/mBio.01371-14. 25028427 PMC4161262

[40] Yatsunenko T, Rey FE, Manary MJ, Trehan I, Dominguez-Bello MG, Contreras M, Magris M, Hidalgo G, Baldassano RN, Anokhin AP, Heath AC, Warner B, Reeder J, Kuczynski J, Caporaso JG, Lozupone CA, Lauber C, Clemente JC, Knights D, Knight R, Gordon JI 2012 Human gut microbiome viewed across age and geography. Nature 486:222–227. doi:10.1038/nature11053. 22699611 PMC3376388

[41] Westcott SL, Schloss PD 2015 De novo clustering methods outperform reference-based methods for assigning 16S rRNA gene sequences to operational taxonomic units. PeerJ 3:e14872. doi:10.7717/peerj.1487. PMC4675110 26664811

[42] Kozich JJ, Westcott SL, Baxter NT, Highlander SK, Schloss PD 2013 Development of a dual-index sequencing strategy and curation pipeline for analyzing amplicon sequence data on the miseq Illumina sequencing platform. Appl Environ Microbiol 79:5112–5120. doi:10.1128/AEM.01043-13. 23793624 PMC3753973

[43] Schloss PD, Gevers D, Westcott SL 2011 Reducing the effects of PCR amplification and sequencing artifacts on 16S rRNA-based studies. PLoS One 6:e27310. doi:10.1371/journal.pone.0027310. 22194782 PMC3237409

