# Supplementary figures and images for: Open-Source Sequence Clustering Methods Improve the State Of the Art
Source: mSystems. 2016 Feb 9;1(1):e00003-15. doi: 10.1128/mSystems.00003-15 (PMC5069751; doi:10.1128/mSystems.00003-15)

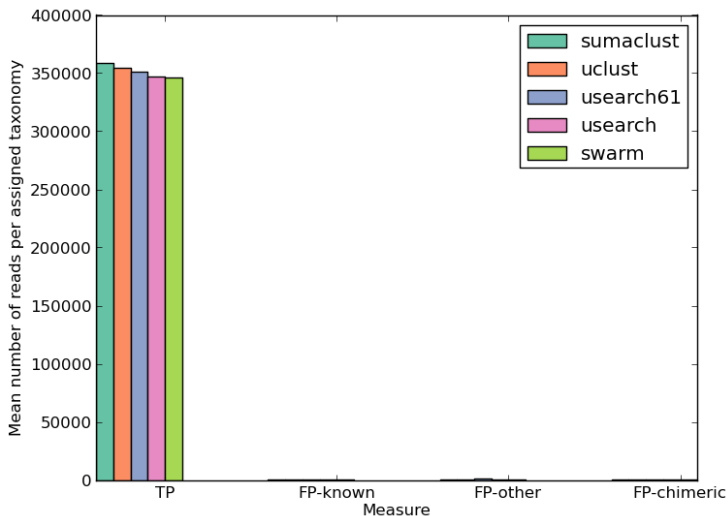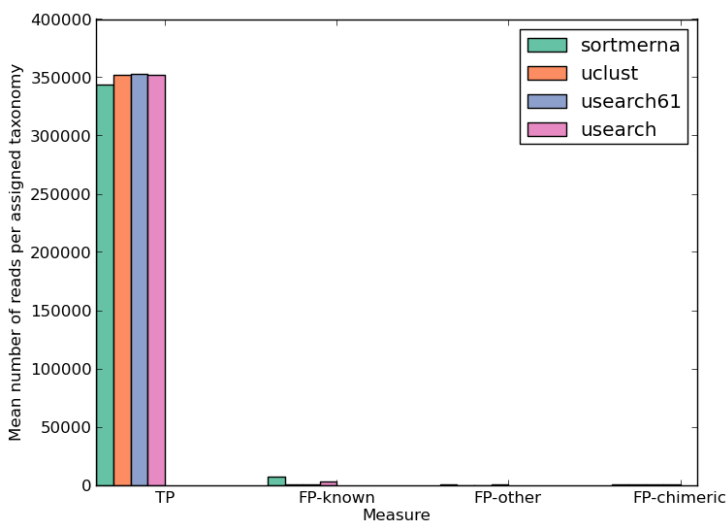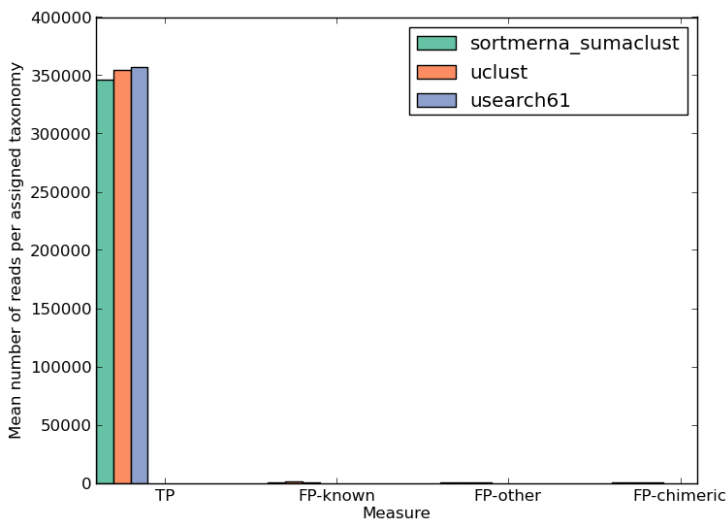

Supplement: Figure S1 [file sys001162002sf1.pdf]

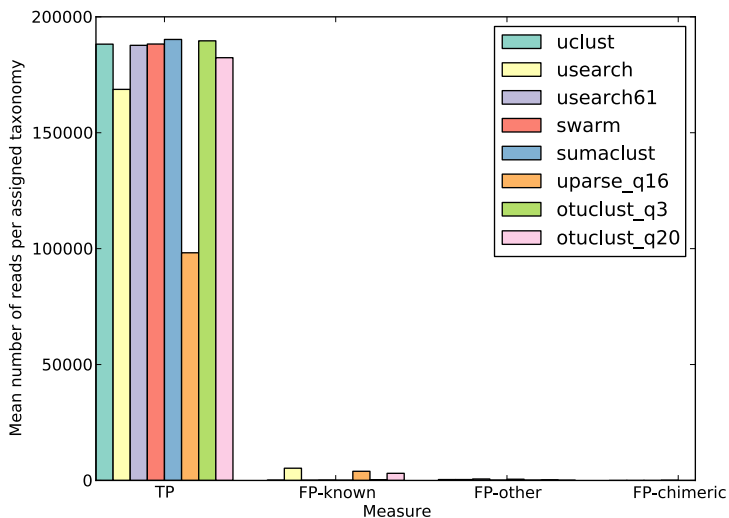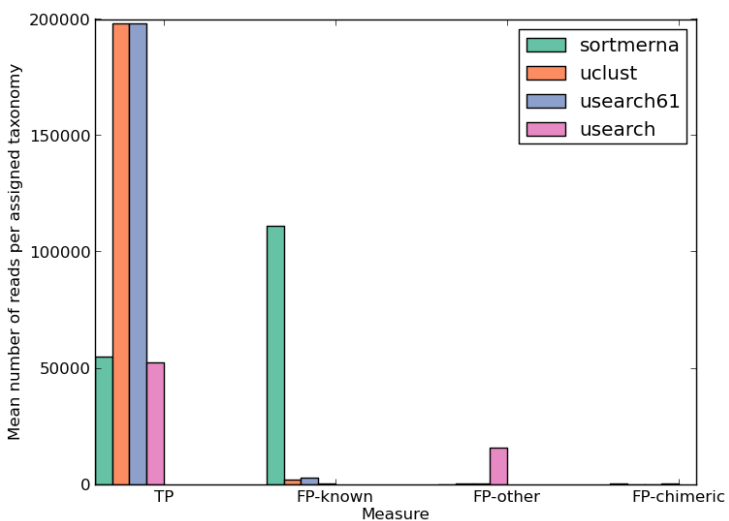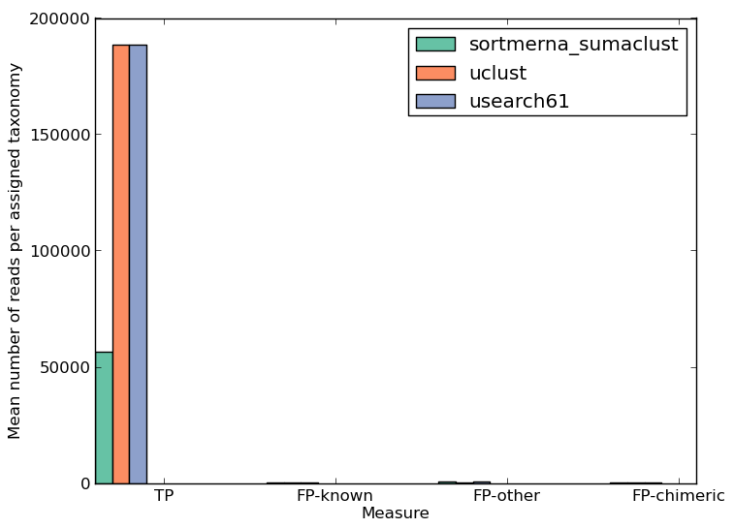

Supplement: Figure S2 [file sys001162002sf2.pdf]

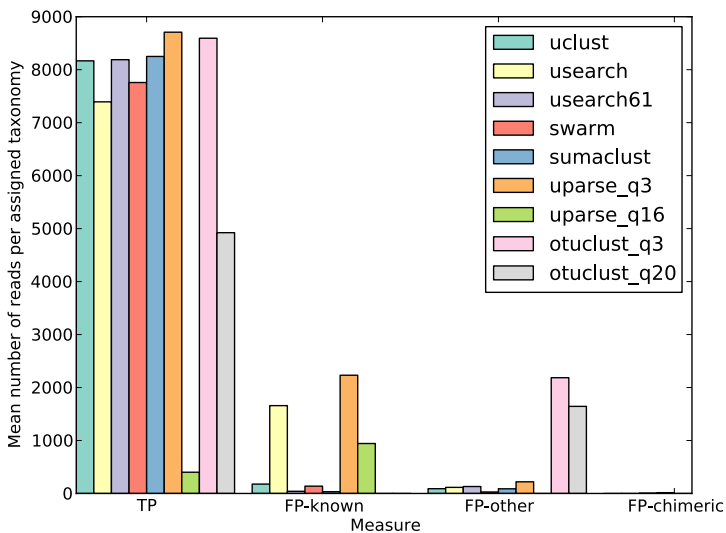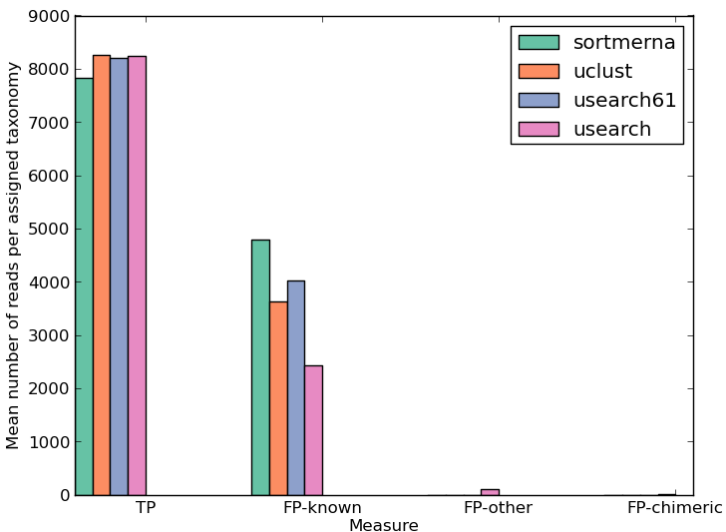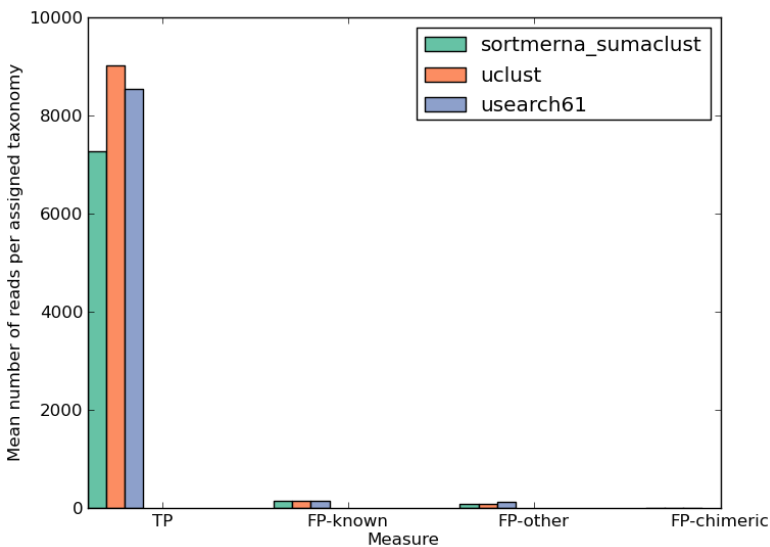

Supplement: Figure S3 [file sys001162002sf3.pdf]

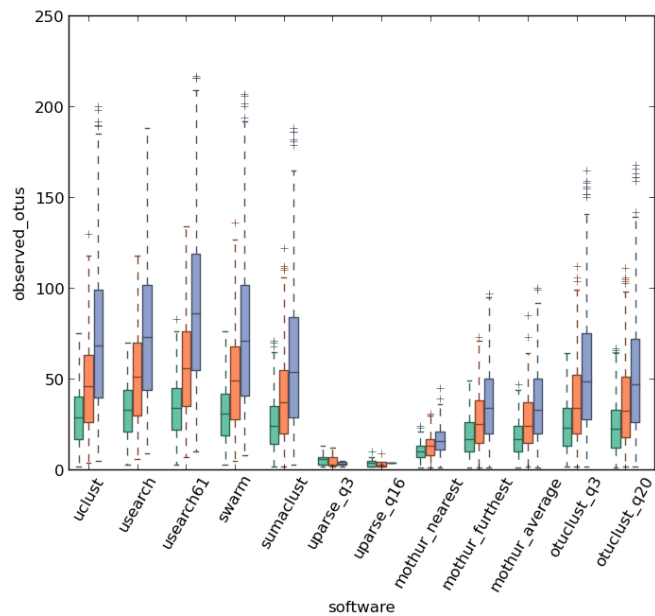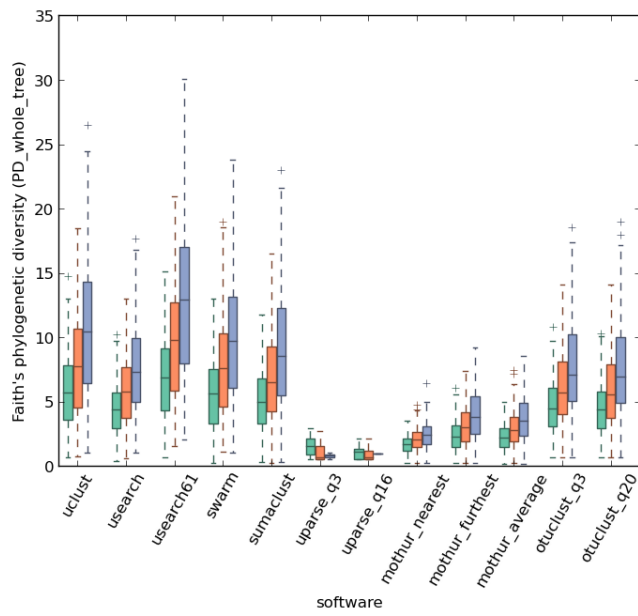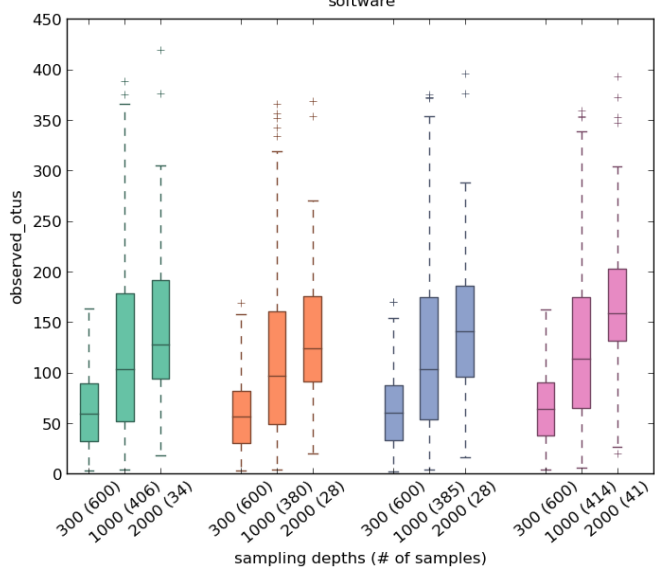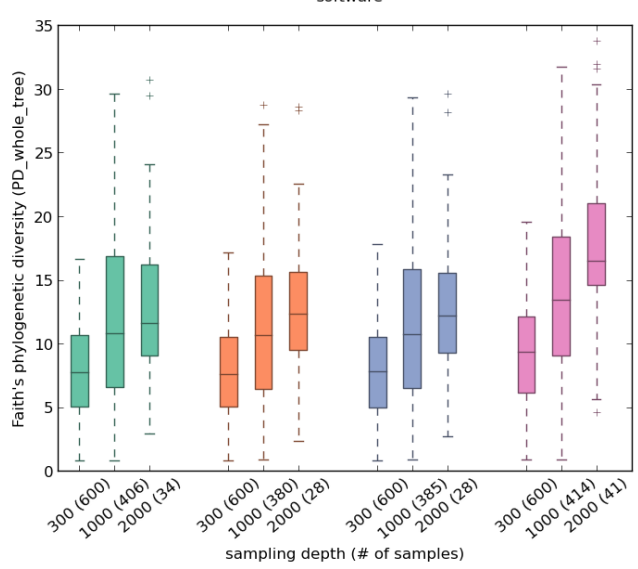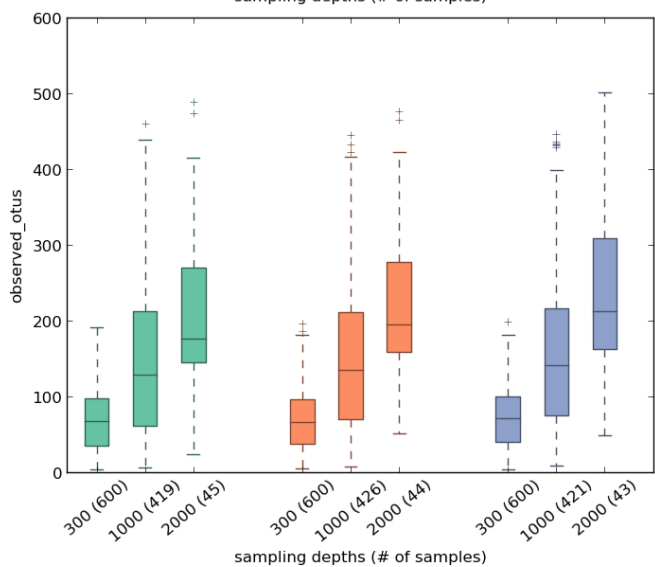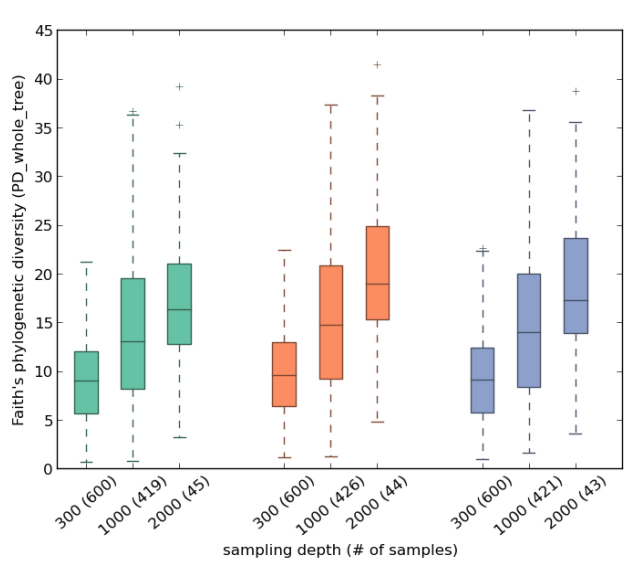

Supplement: Figure S4 [file sys001162002sf4.pdf]

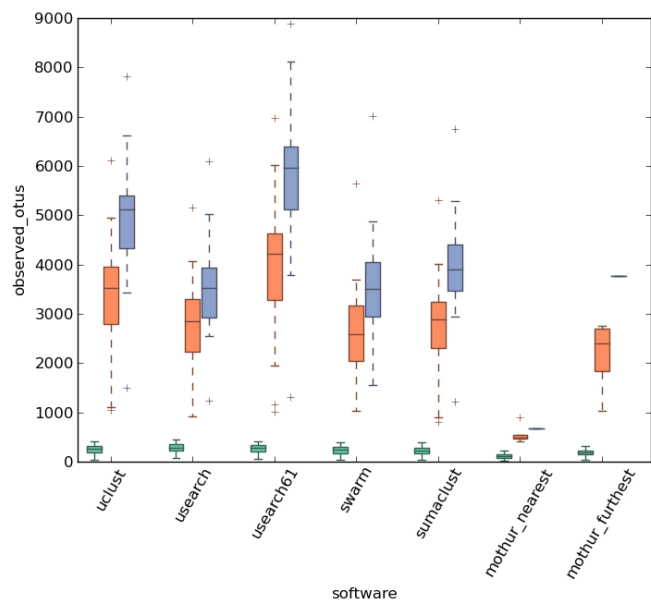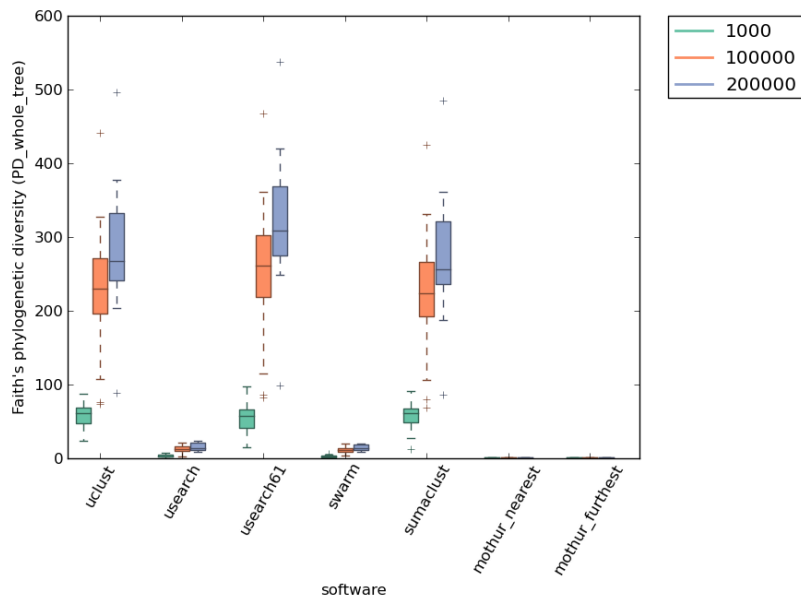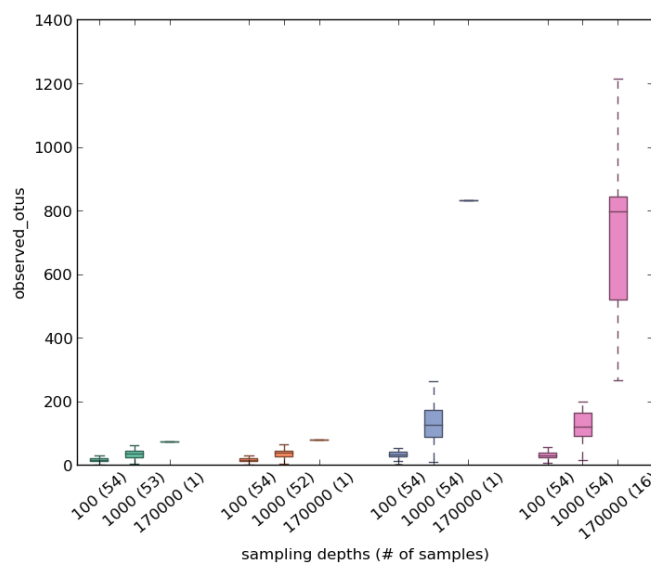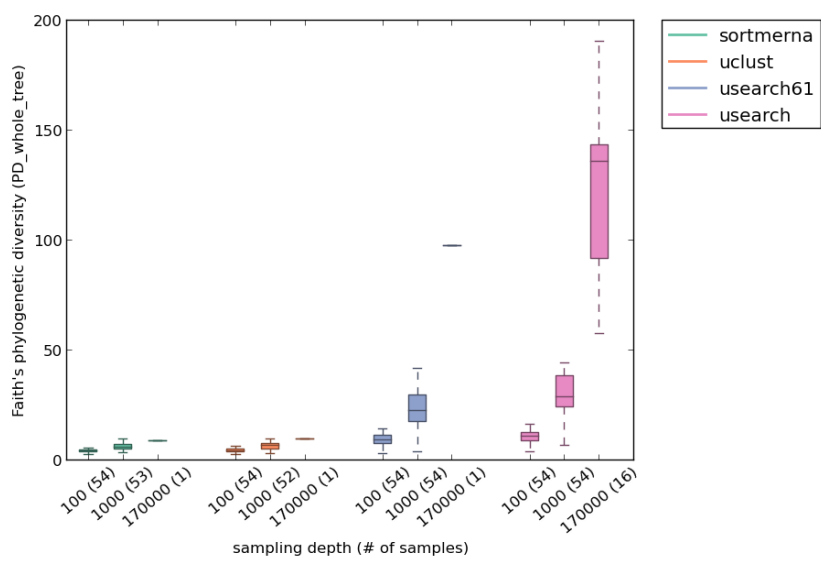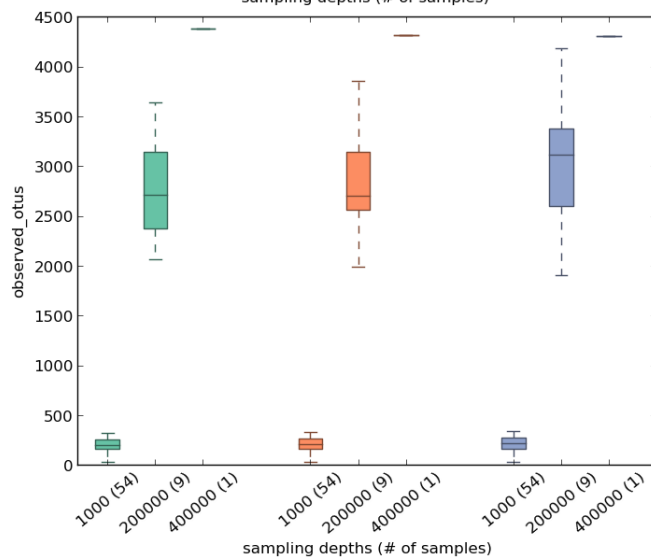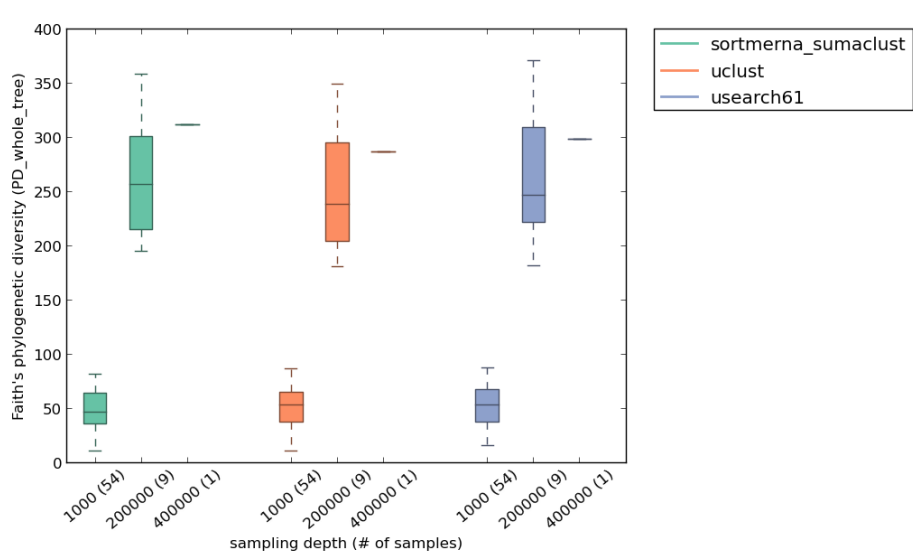

Supplement: Figure S5 [file sys001162002sf5.pdf]
