# Supplementary material for: Open-Source Sequence Clustering Methods Improve the State Of the Art
Source: mSystems. 2016 Feb 9;1(1):e00003-15. doi: 10.1128/mSystems.00003-15 (PMC5069751; doi:10.1128/mSystems.00003-15)
Supplement: Table S1 [file sys001162002st6.pdf]

Software	Download link
QIIME 1.9.0	https://github.com/biocore/qiime/releases/tag/1.9.0
SumaClust 1.0.00	http://metabarcoding.org/sumatra/wiki/download
Swarm 1.2.19	https://github.com/torognes/swarm/releases/tag/1.2.19
SortMeRNA 2.0	https://github.com/biocore/sortmerna/releases/tag/2.0
Uclust 1.2.22q	http://www.drive5.com/uclust/downloads1_2_22q.html
Usearch52 (5.2.236), Usearch61 (6.1), Uparse 7.0.1090	http://www.drive5.com/usearch
OTUCLUST 0.1	https://github.com/compmetagen/micca/archive/v0.2.tar.gz
mothur 1.35.1	https://github.com/mothur/mothur
Blast+ 2.2.29	ftp://ftp.ncbi.nlm.nih.gov/blast/executables/blast+
Blast NT database	ftp://ftp.ncbi.nlm.nih.gov/blast/db/nt.*.tar.gz
ART simulator	http://www.niehs.nih.gov/research/resources/software/biostatistics/art/
**Datasets**	
All QIIME filtered and UPARSE non-filtered datasets	ftp.microbio.me/pub/supplemental_otu_clustering_datasets.tar.gz
