# Supplementary material for: Open-Source Sequence Clustering Methods Improve the State Of the Art
Source: mSystems. 2016 Feb 9;1(1):e00003-15. doi: 10.1128/mSystems.00003-15 (PMC5069751; doi:10.1128/mSystems.00003-15)
Supplement: Table S3 [file sys001162002st8.pdf]

	#Taxon
1	Eukaryota;__Metazoa;__Nematoda;__Chromadorea;__Aphelenchidae
2	Eukaryota;__Metazoa;__Nematoda;__Enoplea;__Trichodoridae
3	Eukaryota;__Metazoa;__Nematoda;__Enoplea;__Dorylaimidae
4	Eukaryota;__Metazoa;__Nematoda;__Enoplea;__Longidoridae
5	Eukaryota;__Metazoa;__Nematoda;__Chromadorea;__Rhabditidae
6	Eukaryota;__Metazoa;__Nematoda;__Chromadorea;__Hoplolaimidae
7	Eukaryota;__Metazoa;__Nematoda;__Chromadorea;__Aphelenchida
8	Eukaryota;__Metazoa;__Nematoda;__Chromadorea;__Anguinidae
9	Eukaryota;__Metazoa;__Nematoda;__Chromadorea;__Plectidae
10	Eukaryota;__Metazoa;__Nematoda;__Chromadorea;__Panagrolaimidae
11	Eukaryota;__Metazoa;__Nematoda;__Chromadorea;__Teratocephalidae
12	Eukaryota;__Metazoa;__Nematoda;__Enoplea;__Prismatolaimidae
