# Supplementary material for: Open-Source Sequence Clustering Methods Improve the State Of the Art
Source: mSystems. 2016 Feb 9;1(1):e00003-15. doi: 10.1128/mSystems.00003-15 (PMC5069751; doi:10.1128/mSystems.00003-15)
Supplement: Table S5 [file sys001162002st10.pdf]

software	OTUs - singletons	P	R	F-measure	TP	FN	FP	FP chimeric	FP known	FP other
de novo										
uparse_q3	29	0.6	0.75	0.66	9	3	6	0	4	2
mothur_furthest	149	0.50	0.75	0.60	9	3	9	0	4	5
usearch61	485	0.45	0.83	0.58	10	2	12	2	1	9
usearch52	148	0.47	0.75	0.58	9	3	10	0	2	8
swarm	408	0.45	0.75	0.56	9	3	11	1	3	7
sumaclust	243	0.4	0.66	0.5	8	4	12	2	3	7
uclust	365	0.4	0.66	0.5	8	4	12	2	2	8
otuclust_q3	47	0.47	0.67	0.55	8	4	9	1	3	5
otuclust_q20	43	0.47	0.67	0.55	8	4	9	1	3	5
mothur_nearest	92	0.44	0.67	0.53	8	4	10	0	5	5
mothur_average	116	0.44	0.67	0.53	8	4	10	0	6	4
closed-reference										
sortmerna	70	0.56	0.75	0.64	9	3	7	0	6	1
uclust	72	0.56	0.75	0.64	9	3	7	0	5	2
usearch61	70	0.53	0.66	0.59	8	4	7	0	5	2
usearch52	50	0.5	0.66	0.57	8	4	8	0	1	7
open-reference										
sortmerna_sumaclust	275	0.47	0.75	0.58	9	3	10	1	2	7
usearch61	328	0.47	0.75	0.58	9	3	10	0	2	8
uclust	319	0.42	0.75	0.54	9	3	12	1	2	9
